# A Call to Action to Address the Burden of Cardiovascular Disease in People with Diabetes

**DOI:** 10.5334/gh.1176

**Published:** 2022-12-19

**Authors:** Karolay Lorenty, Ed Harding, Olive Fenton

**Affiliations:** 1 The Health Policy Partnership, UK; 2 Global Heart Hub, Switzerland

**Keywords:** diabetes, cardiovascular disease, cardio-diabetes, health

## Abstract

In 2022, the Global Heart Hub held a Cardio-Diabetes Think Tank to develop consensus on actions that need to be taken to address the growing burden of diabetes and cardiovascular disease (CVD). Across the world, diabetes affects almost half a billion people, who have a twofold increased risk of CVD. The patient-led think tank brought together the diabetes and CVD communities, with representatives from leading global and regional patient and professional organisations, and produced a report outlining tangible and specific actions that reflect what matters most to patients. The think tank calls for key players in both communities to work together to implement these actions, putting people at the centre of decision-making and improving cardio-diabetes care.

## Major Gaps in Care for People with Diabetes Incur Significant Yet Often Avoidable Burden of CVD

Cardiovascular disease (CVD) is a serious risk in people with diabetes. Yet despite increased understanding of the interrelationship between diabetes and CVD [1,2,3], and clear guidelines for optimal management, everyday clinical practice often does not follow suit. Not only is one in two adults with diabetes unaware of their condition [4], but even after diagnosis, they may be equally unaware of their heightened risk of CVD, and common risk factors are often inadequately managed [4,5,6].

These gaps come at an enormous cost. Diabetes already affects almost half a billion people globally and by 2030 its prevalence is projected to increase by 25% [7]; type 2 diabetes accounts for 90% of these cases [8]. People with diabetes have a twofold increased risk of CVD [9,10], the leading cause of mortality in this group [11]. Treating people who have diabetes and CVD costs two to three times higher than treating those with diabetes alone [11].

In an effort to better understand the gaps in care as well as the structural barriers behind them, the World Heart Federation and the International Diabetes Federation put out a global survey of experts in diabetes and CVD, culminating in *A roadmap on the prevention of cardiovascular disease among people living with diabetes* [6]. They identified a number of barriers, including lack of awareness, limited adherence to clinical guidelines, inadequate communication between healthcare professionals, and lack of access to medicines.

Recognising the need for stronger patient voice in this area, in 2020 the Global Heart Hub brought together representatives of the diabetes and CVD patient communities in a series of round tables. They arrived at a unified consensus of the policy and care gaps in cardio-diabetes, which overlapped with the roadmap, verifying its findings and grounding them into the experience of patients. The round tables resulted in the publication of *Promoting cardiovascular health in people living with, or at risk of, type 2 diabetes*, a report outlining priority areas to focus on [12].

## Defining Priority Actions in Cardio-Diabetes: What Matters Most to Patients?

Building on this work, in May 2022 the Global Heart Hub organised a Cardio-Diabetes Think Tank to define a shortlist of critical advocacy goals and prioritise tangible, consistent demands for decision-makers grounded in the patient experience.

The think tank engaged representatives of global and regional organisations, including four patient groups (Diabetes UK, Pacientes de Corazón, ParSirdi.lv and Diabetes Sisters), three umbrella organisations (Global Heart Hub, World Heart Federation and International Diabetes Federation) and professional societies of nursing, primary and secondary care (Preventive Cardiovascular Nurses Association, International Primary Care Cardiovascular Society, Worldwide Cardiodiabetes).

The participants agreed on four pressing issues which should unite CVD and diabetes advocacy communities and help prioritise the policy demands for to decision-makers:

1. Many governments do not have formal national strategies for diabetes and CVD.
2. There is low public awareness and understanding of the interrelationship between diabetes and CVD, particularly their risk factors and how to address them.
3. Patients are not seen as equal partners in the management of their risk factors and treatment of their conditions.
4. Integrated care delivery, from diagnosis to treatment, is often lacking in many health systems.

The think tank then went further by outlining priority actions to help address these issues on a global and national scale (see Table 1 and the full report, *Cardio-Diabetes Think Tank: call to action* [13]).

**Table 1:** Priority actions for cardio-diabetes advocacy.


PRIORITY ACTION 1: DOCUMENT A CLEAR PICTURE OF THE STATE OF PLAY IN CARDIO-DIABETES

Establish a new cardio-diabetes coalition that puts patients at the centre and makes the case for change to decision-makers at a national level. Undertake an analysis of the state of play in cardio-diabetes policy. Develop a global position statement on quality of care for cardio-diabetes.

**PRIORITY ACTION 2: DEVELOP COMMUNICATION RESOURCES AND CAMPAIGNS TO IMPROVE PUBLIC UNDERSTANDING OF CARDIO-DIABETES RISK AND DISEASE PREVENTION**

Develop communication and educational resources to help both healthcare professionals and patients understand and act on the relationship between diabetes and CVD. Develop public campaigns to raise awareness of the need for a healthy lifestyle and a supportive environment for the prevention of diabetes and CVD.

**PRIORITY ACTION 3: DEVELOP SUPPORT TOOLS TO EMPOWER PATIENTS TO BECOME EQUAL PARTNERS IN THE MANAGEMENT AND TREATMENT OF THEIR CONDITIONS**

Develop resources to help healthcare professionals build stronger partnerships with people living with diabetes at risk of or living with CVD. Develop patient-focused resources to raise awareness of the right to shared decision-making and how to assess the quality of care provided.

**PRIORITY ACTION 4: IMPROVE ACCESS TO AN INTEGRATED CARE PATHWAY FOR CARDIO-DIABETES**

Promote a clear vision for continuity of care across settings. Improve access to specialist-coordinated multidisciplinary teams. Support electronic healthcare records, data sharing, and digitally enhanced and remote care to facilitate truly integrated care.


## Call for the Global Cardio-Diabetes Community to Work Together to a Common Goal

The think tank was able to achieve a modernised, succinct and highly pragmatic consensus for joint advocacy actions between CVD and diabetes stakeholders that address the priorities of patients. The round tables and think tank represent the first time that the patient community led the way in developing tangible advocacy and policy actions in cardio-diabetes, with close input from leading advocacy organisations and specialists.

The consensus put forward by the think tank presents a new opportunity for global clinical and patient communities across diabetes and CVD to work together more closely to a shared agenda, and for individual organisations to take ownership of specific actions and commit to their implementation.

The actions identified will require significant effort, but the think tank consensus also gives hope that a renewed focus across different sectors and disciplines will forge a more defined cardio-diabetes community, with a clear and actionable agenda.

## Think Tank Participants

Carlos Castro, *patient, CEO Pacientes de Corazón, Mexico*

Jean-Luc Eiselé, *CEO, World Heart Federation*

Emma Elvin, *Senior Clinical Advisor, Diabetes UK*

Richard Hobbs, *Chair, European Primary Care Cardiovascular Society; President, International Primary Care Cardiovascular Society; Professor of Primary Care, University of Oxford; Head of Nuffield Department of Primary Care Health Sciences*

Neil Johnson, *Executive Director, Global Heart Hub; CEO Croi, the Heart & Stroke Charity and National Institute for Prevention and Cardiovascular Health, Ireland*

Cindy Lamendola, *Board Member, Preventive Cardiovascular Nurses Association; Nurse Practitioner/Clinical Research Nurse Coordinator, Stanford University School of Medicine*

Inese Maurina, *patient, CEO, ParSirdi.lv, Latvia*

Anna Norton, *patient, CEO, Diabetes Sisters, USA*

Jorge Plutzky, *Board Member, Worldwide Cardiodiabetes; Director, PreventiveCardiology, Brigham and Women’s Hospital; Principal Investigator and AssociateProfessor, Harvard Medical School*

Naveed Sattar, *Board Member, Worldwide Cardiodiabetes; Professor of MetabolicMedicine, University of Glasgow; Honorary Consultant in Metabolic Medicine, Glasgow Royal Infirmary*

Beatriz Yáñez Jiménez, *Advocacy Lead, International Diabetes Federation*

## References

[1] Anonymous. Diabetes mellitus: A major risk factor for cardiovascular disease. A joint editorial statement by the American Diabetes Association; The National Heart, Lung, and Blood Institute; The Juvenile Diabetes Foundation International; The National Institute of Diabetes and Digestive and Kidney Diseases; and The American Heart Association. Circulation. 1999; 100(10): 1132–3. DOI: 10.1161/01.CIR.100.10.1132 10477541

[2] Joseph JJ, Deedwania P, Acharya T, et al. Comprehensive management of cardiovascular risk factors for adults with type 2 diabetes: A scientific statement from the American Heart Association. Circulation. 2022; 145(9): e722–e59. DOI: 10.1161/CIR.0000000000001040 35000404

[3] Rydén L, Standl E, Bartnik M, et al. Guidelines on diabetes, pre-diabetes, and cardiovascular diseases: executive summary. The Task Force on Diabetes and Cardiovascular Diseases of the European Society of Cardiology (ESC) and of the European Association for the Study of Diabetes (EASD). Eur Heart J. 2007; 28(1): 88–136. 17220161 10.1093/eurheartj/ehl260

[4] International Diabetes Federation. Diabetes Atlas. 10th edition. Brussels: IDF; 2021.

[5] Saeedi P, Karuranga S, Hammond L, et al. Cardiovascular diseases and risk factors knowledge and awareness in people with type 2 diabetes mellitus: A global evaluation. Diabetes Research and Clinical Practice. 2020; 165: 108194. DOI: 10.1016/j.diabres.2020.108194 32389743

[6] Mitchell S, Malanda B, Damasceno A, et al. A roadmap on the prevention of cardiovascular disease among people living with diabetes. Glob Heart. 2019; 14(3): 215–40. DOI: 10.1016/j.gheart.2019.07.009 31451236

[7] Saeedi P, Petersohn I, Salpea P, et al. Global and regional diabetes prevalence estimates for 2019 and projections for 2030 and 2045: Results from the International Diabetes Federation Diabetes Atlas, 9th edition. Diabetes Res Clin Pract. 2019; 157: 107843. DOI: 10.1016/j.diabres.2019.107843 31518657

[8] Maahs D, Daniels S, de Ferranti S, et al. Cardiovascular disease risk factors in youth with diabetes mellitus. Circulation. 2014; 130(17): 1532–58. DOI: 10.1161/CIR.0000000000000094 25170098

[9] The Emerging Risk Factors Collaboration. Diabetes mellitus, fasting blood glucose concentration, and risk of vascular disease: A collaborative meta-analysis of 102 prospective studies. The Lancet. 2010; 375(9733): 2215–22. DOI: 10.1016/S0140-6736(10)60484-9 PMC2904878 20609967

[10] Booth GL, Kapral MK, Fung K, et al. 2006. Relation between age and cardiovascular disease in men and women with diabetes compared with non-diabetic people: A population-based retrospective cohort study. Lancet. 2006; 368(9529): 29–36. DOI: 10.1016/S0140-6736(06)68967-8 16815377

[11] Einarson TR, Acs A, Ludwig C, et al. Economic burden of cardiovascular disease in type 2 diabetes: A systematic review. Value Health. 2018; 21(7): 881–90. DOI: 10.1016/j.jval.2017.12.019 30005761

[12] Global Heart Hub. Promoting cardiovascular health in people living with, or at risk of, type 2 diabetes: priorities for collaboration between the diabetes and cardiovascular patient communities. Galway: GHH; 2021.

[13] Global Heart Hub, The Health Policy Partnership. Cardio-Diabetes Think Tank: Call To Action: Advancing action through patient collaboration. Galway: GHH; 2022.

